# A Longitudinal Examination of the Mediating Role of Body Dissatisfaction in the Relationship Between Pornography Use Frequency and Eating Disturbances: A Cross-Lagged Mediation Model

**DOI:** 10.1007/s10508-025-03289-x

**Published:** 2025-11-20

**Authors:** Süleyman Agah Demirgül, Fernando Fernandez Aranda, Susana Jiménez Murcia, Borbála Paksi, Andrea Czakó, Zsolt Demetrovics, Beáta Bőthe

**Affiliations:** 1 Institute of Psychology, ELTE Eötvös Loránd University, Budapest, Izabella U. 46, Budapest, 1064 Hungary; 2 Doctoral School of Psychology, ELTE Eötvös Loránd University, Budapest, Hungary; 3 Trauma Intervention and Research Center/Global and Regional Studies Center, Psychology Department, Necmettin Erbakan University, Konya, Turkey; 5 Psychoneurobiology of Eating and Addictive Behaviors Group, Faculty of Education and Psychology, Neurosciences Programme, Bellvitge Biomedical Research Institute, Barcelona, Spain; 6 University of Barcelona and CIBERobn, Barcelona, Spain; 7 Hospital Universitario de Bellvitge: Hospital Universitari de Bellvitge, Barcelona, Spain; 8 Institute of Education, ELTE Eötvös Loránd University, Budapest, Hungary; 9 Centre of Excellence in Responsible Gaming, University of Gibraltar, Europa Point, Gibraltar; 10 College of Education, Psychology and Social Work, Flinders University, Adelaide, Australia; 11 Département de Psychologie, Université de Montréal, Montreal, QC Canada; 12 Centre de recherche interdisciplinaire sur les problèmes conjugaux et les agressions sexuelles, Montreal, QC Canada

**Keywords:** Pornography, Body dissatisfaction, Eating disturbances, Adults, Longitudinal design

## Abstract

Albeit a positive association between pornography use frequency (PUF) and body dissatisfaction has been identified, studies examining the link between PUF and disordered eating behavior (DEB) have been highly limited in scope (e.g., only focused on men) and have several limitations (e.g., lack of a longitudinal study design and small sample size). The present study aimed to address one of these gaps by examining the longitudinal associations between PUF and DEB, while considering the mediating role of body dissatisfaction over a 1-year period in a sample of young adults. We performed an autoregressive cross-lagged analysis with a multi-group approach among 3764 adults (*M*_*age*_ = 23.00, *SD *_*age*_ = 4.74, 48.24% men and 51.75% women). Our findings showed that higher levels of PUF were cross-sectionally associated with higher levels of DEB among men and women at T1. However, at T2, higher levels of PUF were associated with lower levels of DEB among men and women. Longitudinally, baseline PUF was positively associated with DEB among men and women. However, the reverse association was not observed. Furthermore, the results indicated that body dissatisfaction partially mediated the associations of PUF at baseline (T1) with DEB at the 1-year follow-up (T2) both for men (*b*_indirect_ = .047, 95% CI [.012, .078], *p* = .011) and women (*b*_indirect_ = .033, 95% CI [.001, .059], *p* = .044). Body dissatisfaction seems to play a significant mediating role in the relationship between PUF and DEB. Clinicians treating clients with DEB may consider PUF as a potential contributing factor to the development of eating disturbances via body dissatisfaction.

## Introduction

Technological developments have contributed to pornography use (60–98% report lifetime pornography use) and eating disorders in Western countries (8.4%) (Ballester-Arnal et al., 2023; Galmiche et al., 2019; Ramos-Galarza et al., 2023). In the context of pornography, its increased prevalence can be attributed to its easy access, availability, and affordability, resulting from technological advancements (Cooper, 1998; Rowland & Uribe, 2020). Nonetheless, along with the technological developments the prevalence of eating disorders has also increased due to idealized bodies presented on online platforms (Šmahel et al., 2018). Pornography is one of the platforms that depicts extreme bodies and can lead to physical and emotional changes (Kohut et al., 2020) as well as body dissatisfaction (Paslakis et al., 2022). As pornography typically portrays idealized and exaggerated bodies (Rothman, 2021), some findings suggest an association between pornography use frequency (PUF) and disordered eating behavior (DEB) via body dissatisfaction (Duggan & McCreary, 2013; Gewirtz-Meydan & Spivak-Lavi, 2023; Griffiths et al., 2018).

Previous research has emphasized the potential impact of sociocultural factors on body image concerns and eating disorder symptoms through mechanisms such as social comparison and the internalization of social norms (Bonfanti et al., 2025). The tripartite influence model can be used to explain how various factors can contribute to body dissatisfaction and DEB (Thompson et al., 1999). According to this model, peers, family, and media may all contribute to body dissatisfaction and DEB by encouraging the internalization of societal appearance ideals (Dakanalis et al., 2015) and engagement in social comparison. In the present study, this model provides a framework for understanding the link between PUF and DEB among men and women as it highlights how pornography may relate to individuals’ body image and perceptions of how others evaluate their appearance, which in turn may result in DEB (Rodgers & Chabrol, 2009; Thompson et al., 2004).

Moreover, although the tripartite influence model accounts for the association between prior PUF and later DEB, a reverse association is also possible. Baseline DEB may relate to later levels of PUF. Previous studies have found that excessive pornography use is positively associated with escapism and stress reduction (Bőthe et al., 2021). Given that DEB, particularly overeating and food restriction, are linked to negative affect (Eck & Byrd-Bredbenner, 2021), it is possible that DEB may result in negative emotions, which in turn may drive individuals to engage in greater pornography use to cope with negative emotions associated with their DEB.

All the studies on pornography use and DEBs reviewed so far include several limitations (e.g., focusing on men only, using a small sample, and relying on cross-sectional study designs). Therefore, the current study aimed to examine both cross-sectional and longitudinal associations between PUF and DEB in a sample of young Hungarian adults, examining the mediating role of body dissatisfaction while considering potential gender differences.

### Pornography Use

Pornography lacks a single, unified definition. Some studies have characterized pornography as any written or visual material depicting explicit nudity in the absence of any sexual activity (Wright & Randall, 2012). While some scholars incorporated sexual acts into the definition (Peter & Valkenburg, 2011). However, a comprehensive study suggests that pornography may not always involve sexual acts but may be limited to nudity alone eliciting immediate sexual and emotional reactions. It may also lead to cognitive, emotional, and behavioral alterations (Kohut et al., 2020).

Pornography use is highly prevalent among both men and women. Recent studies conducted on nationally representative samples from Australia (Rissel et al., 2017), Norway (Træen et al., 2004), Poland (Lewczuk et al., 2022), and the United States (Herbenick et al., 2020) aged between 18 and 60, suggested that 70–94% of individuals have engaged in pornography use at some point in their lives. The prevalence of pornography consumption among men exceeds that among women across their entire lifespan. According to a recent study, the frequency of pornography use varied between 46 to 97% among adult men and between 16–38% among adult women (Rissel et al., 2017). Nonetheless, women tend to engage in pornography use more often than men when it is intended to create mutual sexual pleasure through mutual participation (Solano et al., 2020). Additionally, while men tend to use only pornographic videos, women may prefer both visual and written pornography (Solano et al., 2020). Men and women also differ in their preferences for pornographic contexts. Women tend to seek more interactive forms, such as erotic chatting or sexting (Maheux et al., 2021; Mills, 1998), whereas men tend to seek more individual activities, such as pornography watching (Solano et al., 2020).

In addition to being a safe environment for learning about sexuality, a considerable amount of literature has been published on the potential negative consequences of PUF (Singareddy et al., 2025). These studies have emphasized both sexual and mental health-related negative consequences such as diminished interest in sexual activity with one's partner (Poulsen et al., 2013; Stewart & Szymanski, 2012; Sun et al., 2016), low self-esteem (Kvalem et al., 2016), anxiety (Borgogna et al., 2018a, 2018b), depression (Diengdoh & Ali, 2022) as well as marital (Perry & Whitehead, 2019), and occupational problems (Kumar et al., 2021).

### Disordered Eating Behavior

DEBs are eating behaviors that do not fall within the diagnostic criteria for eating disorders. This term includes a variety of behaviors that deviate from typical eating patterns, but do not meet the strict criteria for a formal eating disorder diagnosis (Pennesi & Wade, 2016). These dysfunctional eating patterns include excessive dieting, fasting, vomiting and emotional eating without the presence of binge eating episodes (Croll et al., 2002). Although DEB can differ from clinically diagnosed eating disorders, it may lead to more severe eating-related problems, resulting in clinically diagnosed eating disorders (Bryla, 2003; Hilbert et al., 2014; Killen et al., 1996).

Persistent DEB, if not properly managed, can progressively transform into diagnosable eating disorders. Among these eating disorders the most well-known forms are bulimia nervosa and binge eating. Bulimia Nervosa is characterized by recurrent episodes of binge eating, accompanied by persistent inappropriate compensatory behaviors of an individual’s life (Hayaki, 2009) (e.g., self-induced vomiting and use of laxatives). On the other hand, binge eating is characterized by recurrent episodes of eating large amounts of food within a short period of time (e.g., within any 2-h period) in the absence of any notable efforts towards weight control behavior (Fairburn & Cooper, 2011). In addition to these well-known eating disorders, over the last 2 decades, with the development of technology, food addiction (Bessesen et al., 2008; Gordon et al., 2018) and orthorexia (Demirgul & Rigó, 2023) nervosa have gained significant attention as emerging DEB, although they have not been included in the *Diagnostic and Statistical Manual of Mental Disorders* (DSM-5) (APA, 2013). Regarding food addiction, although there is no agreed upon definition, it refers to specific behaviors related to food that involves excessive and uncontrolled consumption of high-calorie foods despite negative consequences (Imperatori et al., 2016; Schulte et al., 2017). Therefore, individuals with food addiction are more likely to consume processed food that are rich in sugar, salt, and fat (Schulte et al., 2019; Gearhardt et al., 2009). Additionally, refined carbohydrates trigger a similar increase in extracellular dopamine levels in the brain therefore it shares some similar neurological features with other addictive substances like nicotine or alcohol (De Luca, 2014; Di Chiara & Imperato, 1988; Gearhardt et al., 2023). Based on a recent systematic review, it has been reported that the overall estimated pooled prevalence of food addiction among adults is approximately 14% (Praxedes et al., 2022). In contrast, orthorexia nervosa is characterized by the pathological fixation on healthy eating (Dunn & Bratman, 2016) individuals with orthorexia nervosa devote excessive time to selecting, preparing, and consuming healthy food, resulting in significant impairment in one’s social and interpersonal life (Lopes et al., 2020; Moroze et al., 2015).

The onset of eating disorders typically occurs during adolescence and young adulthood (Halmi, 2005). The National Comorbidity Survey Replication (NCS-R) states that the onset of different types of eating disorders can vary between 18 and 21 years of age. Additionally, a study on clinical samples of various eating disorders (i.e., Anorexia Nervosa and Bulimia Nervosa) found evidence consistent with NCS-R findings, indicating that the typical age of eating disorder onset is around 18 years old (Volpe et al., 2016). The prevalence rates of eating disorders differ based on the type of eating disorders and gender, with a higher rate observed among women. For instance, the prevalence of bulimia nervosa in women is 3%, whereas that in men is only 1% (van Eeden et al., 2021). Similarly, the prevalence of binge eating is 13% in women and 5%in men (Mustelin et al., 2017). Moreover, the prevalence of food addiction is 6% in women and 3% in men (Pedram et al., 2013). In summary, eating disorders exhibit significant gender disparities and frequently co-occur with body-related issues, resulting in substantial impairment of an individual's life.

Beyond sociocultural influences, the etiology of DEB is recognized as multifactorial, including biological, psychological, and developmental components (Collier & Treasure, 2004). Biologically, prior research has underscored the role of serotonin, a neurotransmitter critically involved in appetite regulation and emotional regulation (Compan et al., 2012). Psychologically, personality traits, such as perfectionism (Danielsen et al., 2023), neuroticism, and obsessive–compulsive tendencies have been positively associated with DEB (Cassin & von Ranson, 2005). From a developmental perspective, early life experiences, particularly childhood sexual abuse have been identified as significant risk factors that may contribute to the onset of DEB in later life (Fischer et al., 2010).

### Associations Between Pornography Use and Disordered Eating Behavior

Besides biological, psychological, and developmental factors contributing to the DEB a core issue in eating disorders is predominantly centered on physical concern, such as body surveillance (Martin-Wagar & Weigold, 2023) and body dissatisfaction (Fairburn & Cooper, 2011). Moreover, as mentioned above sociocultural factors, particularly mass media, may have a significant impact on body image concerns. Traditional media, including magazines and TV, often promote unrealistic body ideals, such as extremely thin and toned figures, resulting in preoccupation with weight loss (Groesz et al., 2002). Similar to other forms of media, pornography also portrays idealized bodies, using actors whose body features are not representative of the general population in terms of height, muscularity, and size of genitalia (Leickly et al., 2017). As a result, the urge to conform to societal ‘standards’ for ‘beauty’ gained through either mass media or pornography may result in DEB by changing the eating habits of the individual or through body dissatisfaction (Harriger et al., 2022; Marks et al., 2020). Therefore, scholars have suggested that pornography use may skew an individual’s perceived body image (Borgogna et al., 2018a, 2018b; Demirgül et al., 2025; Duggan & McCreary, 2013; Leickly et al., 2017; Peter & Valkenburg, 2014; Whitfield et al., 2018). For example, pornography use may lead to greater body monitoring tendencies (Doornwaard et al., 2014), internalization of body ideals (Sevic et al., 2020), and a desire for muscularity (Morrison et al., 2007; Tylka & Kroon Van Diest, 2015).

According to research, specific circumstances can heighten the likelihood of DEB (Striegel-Moore et al., 1986) through body dissatisfaction (Karchynskaya et al., 2022; Mazzeo & Bulik, 2009). Objectification Theory posits that prolonged exposure to sexual objectification may result in women internalizing such messages and starting to look at their bodies from an external observer and perceive their own bodies as objects to be examined by other individuals (Fredrickson & Roberts, 1997).Those who hold this perspective are found to be more likely to monitor their bodies resulting in body dissatisfaction (Maheux et al., 2021; Tiggemann & Lynch, 2001). Previous studies have shown that men can also experience self-objectification, albeit to a lesser degree than women with desire for a more muscular body (Daniel et al., 2014; Michaels et al., 2013). This is because men's bodies are objectified as well, although not to the same extent as women's. Body dissatisfaction which results from self-objectification may also contribute to DEB. A previous meta-analytic study has shown that self-objectified women and men experience DEB with women reporting greater DEB than men (Schaefer & Thompson, 2018). Therefore, pornography can also result in self-objectification and DEB due to experiencing body dissatisfaction. Significant gender differences can also be observed as women report greater body checking and DEB than men (Striegel-Moore et al., 2009) in relation to their PUF.

In addition to self-objectification being a significant risk factor for DEB (Tolosa-Sola et al., 2019), they also exhibit a close association with sexual dysfunction along with DEB (Tolosa-Sola et al., 2019). Given that body-related problems may also be associated with pornography consumption and sexual dysfunction (Woertman & Van Den Brink, 2012), some studies examining body-related problems associated with frequent pornography use have included DEB (Duggan & McCreary, 2013; Griffiths et al., 2018; O’Brien et al., 2015; Tylka & Calogero, 2019). However, because body dissatisfaction frequently precipitates DEB (Lladó & González-Soltero, 2017; Tylka, 2004), body dissatisfaction needs to be considered (Duggan & McCreary, 2013; Griffiths et al., 2018). However, among previous studies examining pornography use and DEB, only one study considered the potential role of body dissatisfaction, but that study focused on problematic pornography use (i.e., uncontrolled pornography use resulting in significant distress and functional impairment) rather than PUF in general (Gewirtz-Meydan & Spivak-Lavi, 2023).

Recent studies have observed a link between PUF and DEB; however, findings are not yet fully generalizable, as only a limited body of evidence has been gathered. A recent study using cross-sectional data from Israeli gay and heterosexual men found a positive association between problematic pornography use and DEB (Gewirtz-Meydan & Spivak-Lavi, 2023). Similarly, another study examined the association between PUF and DEB in Australian sexual minority men and showed a weak positive association between PUF and DEB (Griffiths et al., 2018). Zhou’s (2019) study examining contributing factors to DEB by relying on social media communication revealed a positive association between pornography use and DEB. Although some studies have established a positive link between PUF and DEB in men (Gewirtz-Meydan & Spivak-Lavi, 2023; Griffiths et al., 2018), a study comparing gay and heterosexual men’s PUF and DEB observed non-significant associations (Duggan & McCreary, 2013).

Overall, associations between pornography use and DEB is not clear as previous studies yielded inconsistent findings. Moreover, the existing literature on PUF and DEB is not without limitations. For example, all studies used cross-sectional designs, limiting the ability to determine the directionality between PUF and DEB. Additionally, prior studies have primarily focused on men (Gewirtz-Meydan & Spivak-Lavi, 2023), with a small sample size and a predominance of sexual minority men (Duggan & McCreary, 2013; Griffiths et al., 2018). Moreover, a recent study included only Israeli men, where obligatory military service can have an effect on individuals’ views on masculinity, which may result in more DEB (Bartlett & Mitchell, 2015; Gewirtz-Meydan & Spivak-Lavi, 2023; Hudson et al., 2007). In addition, the inclusion of self-selected participants (Duggan & McCreary, 2013) and the absence of large samples (Duggan & McCreary, 2013; Gewirtz-Meydan & Spivak-Lavi, 2023; Griffiths et al., 2018; O’Brien et al., 2015) may introduce bias and limit the generalizability of previous findings. Moreover, body dissatisfaction is one of the most important risk factors of DEB and previous studies established a link between pornography use and body dissatisfaction (Paslakis et al., 2022). Yet, the potential mediating role of body dissatisfaction between PUF and DEB has not been examined yet. Due to these limitations, the current body of research is insufficient to draw conclusions about the association between PUF and DEB, especially among women, who are at a higher risk of developing DEB than men (Hudson et al., 2007).

### The Present Study

We aimed to examine the cross-sectional and longitudinal associations between PUF and DEB in a sample of young Hungarian adults, while examining the mediating effect of body dissatisfaction. Moreover, this study aimed to explore potential differences between men and women on PUF and DEB, given that prior studies did not include women, limiting our understanding (Duggan & McCreary, 2013; Gewirtz-Meydan & Spivak-Lavi, 2023; Griffiths et al., 2018). Our first hypothesis was that PUF would be positively associated with DEB cross-sectionally and longitudinally as well. Our second hypothesis was that women would report higher level of DEB symptoms than men in relation with their PUF. Our third hypothesis was that body dissatisfaction would mediate the association between T1 PUF and T2 DEB.

## Method

### Participants and Procedure

We used data from the Budapest Longitudinal Study (BLS). The BLS is a longitudinal study that examines the factors associated with various addictions and problematic behaviors, such as gaming disorder, gambling disorder, compulsive sexual behavior, and other substance addictions (e.g., alcohol and exercise) among young adults aged 18–34 in Budapest, Hungary.

The data for the first and second waves were acquired between March and July 2019 and June and September 2020, respectively. In both waves, we gathered sociodemographic information along with PUF and DEB. The final sample included 3910 participants. In our study, we included 3764 participants, as they were the only individuals who could be matched across both data collection waves (the remaining participants could not be matched because of issues such as non-matching identification numbers). A stratified random sampling technique was implemented to differentiate participants by age group and neighborhood. Informed consent was obtained prior to data collection. The analysis codes were uploaded in Open Science Framework (OSF; https://shorturl.at/NqcMc). However, due to the confidential nature of the information and the fact that participants were not informed about the potential for their data to be shared publicly, we chose not to upload the dataset to OSF.

A total of 3764 participants completed the survey (M_age_ = 23 years, SD = 4.74). Regarding gender, 1815 (52%) were men and 1949 (48%) were women. Regarding marital status, 2103 (56%) reported being single, 1504 (40%) were married, 10 were widows (0.26%), and 86 (2%) were divorced. The descriptive statistics are presented in Table 1.

**Table 1:** Sociodemographic characteristics of participants

Characteristics	*N*	%	*M*	*SD*
General population	3764	100		
Gender				
Men	1815	51.78		
Women	1949	48.21		
Marital Status				
Single	2103	55.87		
Married	1504	39.95		
Widow	10	0.26		
Divorced	86	2.28		
Did not respond	61	1.62		
Age				
23–29	957	25.42	23	4.74
29–33	1133	30.10		
34–39	1674	44.47		
Highest level of education				
Primary school	3	1		
Vocational school 1–3 grades	144	3.8		
Vocational school 4–5 grades	779	20.5		
Vocational high school	1161	30.5		
High school	701	18.4		
Higher vocational education after high school graduation,	291	7.7		
Higher vocational education, higher technical school (not college)	114	3.0		
College, BA/BSc education	425	11.2		
College, MA/MSc or undivided (integrated) education	128	3.4		
Post-graduated education, doctoral school (PhD, DLA)	18	4		

*M*, mean; *SD*, standard deviation; N, sample size; %, percentage

### Measures

#### Sociodemographic Information

Demographic information, including gender, age, highest level of education, and marital status, was collected and is presented in Table 1.

#### Frequency of Pornography Use

Participants indicated the past-year PUF with the question, “During the last year, how often did you watch pornographic videos/films?” on a 10-point scale (0 = “not once in the last year," 10 = "more than seven times a week”).

#### Body Dissatisfaction

Body dissatisfaction was assessed using the subscale of the Body Attitude Test (BAT; Probst et al., 1995). This subscale comprises four items rated on a five-point Likert scale ranging from 1 (*never)* to 5 (*always).* An example item is “When I look at myself in the mirror, I am dissatisfied with my own body.” In the present study, the internal consistency (Cronbach’s alpha) of this subscale was α = .84 for T1, and α = .92 for T2. Higher scores on the scale indicate higher body dissatisfaction.

#### Disordered Eating Behavior

Eating disturbances were assessed using the SCOFF questionnaire (Morgan et al., 1999). This instrument consists of five items that use a two-point scale, with respondents indicating either ‘yes’ or ‘no’ for each item. A score of ≥ 2 suggests a strong likelihood of anorexia nervosa or bulimia, with one point awarded for each affirmative response. An example item is, “Would you say food dominates your life?”. In the present study, the internal consistency (Cronbach’s alpha) of this scale was α = .74 for T1, and α = .71 for T2. Higher scores on the scale indicate higher levels of potential DEB.

### Data Analysis

We used SPSS 26 to calculate the descriptive statistics, Cronbach's alpha, and McDonald's omega. Although Cronbach's alpha is often used as a measure of reliability, it is not considered to be the optimal choice (Hayes & Coutts, 2020). In this study, we evaluated the reliability using two methods: McDonald's omega and Cronbach's alpha. Table 2 presents the results. The final, weighted sample comprised 3,733 individuals. Regarding the DEB scale at Time 1, our sample consisted of 3,733 participants, whereas at Time 2, the number of individuals dropped to 2,667, reflecting a 30% attrition rate between the two time points. With regard to the PUF, 3,472 and 2,620 individuals completed the questionnaire at Time 1 and Time 2, respectively, indicating a 24% attrition rate between the two time points. To address the issue of missing data, we employed the Full Information Maximum Likelihood method, as recommended by Enders and Bandalos (2001) and Newman (2014).

**Table 2:** Reliability indices, comparisons of men’s and women’s pornography use frequency, body dissatisfaction, and disordered eating behavior

Variables	Range	ω	α	Total sample	(1) Men	(2) Women	Cohen’s *d*	*p*
				(N = 3764)	(n = 1815)	(n = 1949)		
				*M* (SD)	*M* (SD)	*M* (SD)		
1. Disordered eating behavior^b^	0–2	.74	.73	0.06 (0.16)	0.05 (0.15)	0.07 (0.18)	0.17	< 0.01
2. Disordered eating behavior^b^	0–2	.71	.81	0.07 (0.21)	0.07 (0.22)	0.08 (0.20)	0.21	< 0.01
3. Body dissatisfaction T1^c^	0–4	.84	.84	1.07 (0.02)	0.80 (0.94)	1.27 (1.21)	0.43	< 0.01
4. Body dissatisfaction T2^c^	0–4	.92	.92	0.89 (0.02)	0.77 (1.04)	0.98 (1.13)	0.19	< 0.01

^a^0 = not once in the last year, 1 = 1 time, 2 = 2–6 times last year, 3 = 7–11 times last year, 4 = 1 time per month, 5 = 2–3 times a month, 6 = 1 time per week, 7 = 2–3 times a week 8 = 4–5 times a week, 9 = 6–7 times a week, 10 = more than 7 times a week

^b^1 = yes, 2 = No, c = 0 = never, 1 = rarely, 2 = sometimes, 3 = often, 4 = usually, 5 = always

The primary analysis was conducted using MPlus 8.7, which employed the Robust Maximum Likelihood Estimator (MLR) to examine auto-regressive cross-lagged models and explore the relationship between PUF and DEB. While analyzing the data, we standardized all the measures and tested the mediation model to determine whether the direct and indirect effects were significant by employing the bootstrap method with 10,000 replicate samples along with the calculation of 95% confidence intervals. To test the adequacy of the model fit, we used commonly used goodness of fit indices (Brown, 2015), including the Comparative Fit Index (CFI; ≥ 0.90 for acceptable; ≥ 0.95 for excellent), Root-Mean-Square Error of Approximation (RMSEA; ≤ 0.06 for good, ≤ 0.08 for acceptable), and Tucker Lewis Index (TLI; ≥ 0.95 for good, ≥ 0.90 for acceptable (Brown, 2015). We employed the Full Information Maximum Likelihood method to address missing data (Enders & Bandalos, 2001; Newman, 2014).

We conducted two cross-lagged panel models. Our first model estimated the associations between PUF and DEB and our second model estimated the mediating role of body dissatisfaction between PUF and DEB. Both models tested for potential gender differences. In our first model, we examined the associations between PUF and DEB (Model A) and subsequently, we introduced the gender grouping variable (i.e., men vs. women) into the model and used a multigroup analysis to measure potential differences across genders (i.e., men vs. women) (Model B). Afterward, all associations between PUF and DEB were constrained across gender groups (Model C). Lastly, we examined the differences between Model B and C (i.e., unconstrained and constrained models) to determine whether gender differences were significant, by examining changes in chi-square, CFI, TLI, and RMSEA values. A significant corrected chi-square difference test, significant decreases in CFI and TLI (ΔCFI ≤ 0.010; ΔTLI ≤ 0.010), and significant increases in RMSEA (ΔRMSEA ≤ 0.015) (Bőthe et al., 2021; Chen, 2007; Cheung & Rensvold, 2002) indicated whether the constrained and unconstrained models differed significantly (i.e., whether the associations differed significantly between men and women).

For the mediational model, we examined the indirect effects of PUF on DEB via body dissatisfaction. The mediated effect (ab) of PUF at T1 on DEB at T2 via body dissatisfaction at T2 was defined as the product of Path a (the effect of PUF at T1 on body dissatisfaction at T2) and Path b (the effect of body dissatisfaction at T2 on DEB at T2). Similarly, the mediated effect (cd) of PUF at T1 on DEB at T2 through body dissatisfaction was defined as the product of Path c (the effect of PUF at T1 on Body dissatisfaction). We only examined the mediated effect of body dissatisfaction for one path which is from T1 PUF to T2 DEB (Model D). We then introduced the gender grouping variable (i.e., men vs. women) into the model and used a multigroup analysis to measure potential differences across genders (i.e., men vs. women) in the relationship between PUF and DEB (Model E). Then, all associations between PUF and DEBs were constrained across gender groups (Model F). Finally, we examined the differences between Model E and F (i.e., unconstrained and constrained models) to determine whether gender differences were significant, by examining changes in chi-square, CFI, TLI, and RMSEA values. All the model fit indices are presented in Table 4.

## Results

### Descriptive Statistics of Pornography Use, Body Dissatisfaction, and Disordered Eating Behavior and Comparisons of Men and Women

We conducted an independent-samples t-test to investigate gender-based differences in PUF, DEB, and body dissatisfaction. We observed significant gender differences in PUF, DEB, and body dissatisfaction. According to our findings, men exhibited significantly higher levels of T1 and T2 PUF (T1, *M* = 2.45, *SD* = 2.59, T2, *M* = 1.80, *SD* = 2.81) than women (T1, *M* = 0.98, *SD* = 2.03; T2, *M* = 0.70, *SD* = 1.80). Conversely, women exhibited significantly higher levels of T1 and T2 body dissatisfaction (T1, *M* = 1.27, *SD* = 1.21, T2, *M* = 0.98, *SD* = 1.13) than men (T1, *M* = 0.80, *SD* = 0.94, T2, *M* = 0.77, *SD* = 1.04). Concerning DEB, similarly women exhibited significantly higher levels of T1 and T2 DEB (T1, *M* = 0.08, *SD* = 0.18, T2, *M* = 0.08, *SD* = 0.20) than men (T1, *M* = 0.05, *SD* = 0.15, T2, *M* = 0.07, *SD* = 0.22) see Table 2.

The correlations among the study variables are presented in Table 3. Pearson correlation coefficients showed that associations between PUF and DEB were significant at T1 but not at T2. Additionally, body dissatisfaction was positively associated with PUF at T1 and T2 (rs ranging between .09 to.11, *p* < .001). Moreover, body dissatisfaction was positively associated with DEB at T1 and T2 (rs ranging between .12 to.55, *p* < .001).

**Table 3:** Descriptive statistics, correlation between pornography use frequency, disordered eating behavior, and body dissatisfaction

Variables	(Skew) (SE)	Kurt (SE)	Range	M (*SD*)	1	2	3	4	5	6
1. Pornography use frequency T1^a^	2.93 (0.04)	6.62 (0.08)	0–10	1.60 (2.33)	1					
2. Pornography use frequency T2^a^	2.62 (0.04)	6.07 (0.09)	0–10	0.70 (1.77)	.15**	1				
3. Disordered eating T1^b^	3.01 (0.04)	9.28 (0.09)	0–2	0.06 (0.16)	.12**	.03	1			
4. Disordered eating T2^b^	3.11 (0.04)	9.25 (0.09)	0–2	0.08 (0.21)	.19**	− .01	.17**	1		
5.Body dissatisfaction T1^c^	0.99 (0.04)	0.56 (0.09)	0–4	1.07 (0.02)	.11**	.01	.50**	.12**	1	
6.Body dissatisfaction T2^c^	1.23 (0.04)	0.96 (0.09)	0–4	0.89 (0.21)	.09**	.10**	.26**	.55**	.25**	1

^a^0 = not once in the last year, 1 = 1 time, 2 = 2–6 times last year, 3 = 7–11 times last year, 4 = 1 time per month, 5 = 2–3 times a month, 6 = 1 time per week, 7 = 2–3 times a week 8 = 4–5 times a week, 9 = 6–7 times a week, 10 = more than 7 times a week

^b^1 = yes, 2 = No; c = 0 = never, 1 = rarely, 2 = sometimes, 3 = often, 4 = usually, 5 = always

*p < .05; **p < 0.01

### Cross-Sectional and Longitudinal Associations Between Pornography Use and Disordered Eating Behavior Without the Mediating Variable

Model A showed an excellent fit to the data (see Table 4 for details). To examine whether association between PUF and DEB differed across genders (i.e., men and women), we compared the Model B to the Model C (ΔCFI = 0.172; ΔTLI = 0.286; ΔRMSEA = − 0.048). Moreover, the corrected chi-square difference test result was significant (Δχ^2^ = 32.085; *p* < .001), suggesting significant gender differences.

**Table 4:** Examination of the relationship between pornography use frequency and disordered eating behavior and the mediating role of body dissatisfaction between pornography use frequency and disordered eating behavior

Models	*χ*^2^ (df)	CFI	TLI	RMSEA	RMSEA (90% CI)
Model A: Fully saturated model, no mediating variable (total sample)	0.000 (0)	1.000	1.000	0.000	0.000–0.000
Model B: Fully saturated model no mediating variable, with grouping by gender	0.000 (0)	1.000	1.000	0.000	0.000–0.000
Model C: Same as Model B, parameters constrained to be equal between groups	32.085 (6)	0.828	0.714	0.048	0.033–0.065
Model D: Same as Model A, with body dissatisfaction as a mediator	0.000 (0)	1.000	1.000	0.000	0.000–0.000
Model E: Same as Model D with grouping by gender	0.000 (0)	1.000	1.000	0.000	0.000–0.000
Model F: Same as Model E, parameters constrained to be equal between groups	714.74 (24)	0.309	0.309	0.124	0.116–0.132

χ^2^, Chi-square test; df, degrees of freedom; CFI, comparative fit index; TLI, Tucker–Lewis Index; RMSEA, root-mean-square error of approximation; 90% CI, 90% confidence interval of RMSEA

Among men, higher levels of T1 DEB were associated with higher levels of T1 PUF *(r* = .13, 95% CI [.08, .19],* p* < .001). Higher levels of T2 DEB were associated with lower levels of T2 PUF *(r* = -.08, 95% CI [-.12, -.03],* p* = .001). Longitudinally, higher levels of T1 PUF were associated with higher levels of T2 PUF (*β* = .13, 95% CI [.06, .22],* p* = .001) and higher levels of T1 DEB were associated with higher levels of T2 DEB (*β* = .13, 95% CI [.06, .20],* p* < .001). T1 DEB was not significantly associated with T2 PUF (*β* = .05, 95% CI [-.05, .09],* p* = .104). Higher levels of T1 PUF were associated with higher levels of T2 DEB (*β* = .15, 95% CI [.09, .24],* p* < .001). All significant associations between DEB and PUF had weak effect sizes.

Among women, higher levels of T1 DEB were associated with higher levels of T1 PUF *(r* = .20, 95% CI [.15, .26],* p* < .001). Higher levels of T2 DEB were associated with higher levels of T2 PUF *(r* = .06, 95% CI [.01, .12],* p* = .029). Longitudinally, T1 PUF was not significantly associated with T2 PUF (*β* = −.03, 95% CI [-.06, .07],* p* = .133). T1 DEB was not significantly associated with T2 PUF (*β* = .06, 95% CI [-.02, .15],* p* = .184). Higher levels of T1 PUF were associated with higher levels of T2 DEB (*β* = .21, 95% CI [.06, .24],* p* = .001). Higher levels of T1 DEB were associated with higher levels of T2 DEB (*β* = .11, 95% CI [.03, .17],* p* = .005). All significant associations between DEB and PUF had weak effect sizes. Although our longitudinal analysis provided evidence of directionality, it is important to emphasize that we cannot draw conclusions regarding causal relationships between the study variables.

### The Mediating Role of Body Dissatisfaction in the Associations of Pornography Use and Disordered Eating Behavior

To examine the mediating role of body dissatisfaction (T2) in the relationship between PUF and DEB differed across genders (i.e., men and women), we compared the Model F to the Model E (ΔCFI = 0.691; ΔTLI = 0.691; ΔRMSEA = − 0.124). The corrected chi-square difference test result was significant (Δχ^2^ = 125.438; *p* < .001). These results indicated that when body dissatisfaction was included as a mediating variable, the model differed significantly between men and women. Therefore, the results of Model E are reported in the following section.

Among men, higher levels of T1 DEB were associated with higher levels of T1 PUF *(r* = .13, 95% CI [.08, .19],* p* < .001). Higher levels of T1 DEB were associated with higher levels of T1 body dissatisfaction *(r* = .40, 95% CI [.34, .45],* p* < .001). Higher levels of T1 PUF were associated with higher levels of T1 body dissatisfaction *(r* = .21, 95% CI [.15, .26],* p* < .001). Higher levels of T2 DEB were associated with lower levels of T2 PUF *(r* = −.12, 95% CI [− .17, − .07],* p* < .001). Longitudinally, higher levels of T1 PUF were associated with higher levels of T2 PUF (*β* = .12, 95% CI [.06, .18],* p* < .001). T1 DEB was not significantly associated with T2 DEB (*β* = .03, 95% CI [− .02, .08],* p* = .261). T1 DEB was not significantly associated with T2 PUF (*β* = .23, 95% CI [− .64, 1.47],* p* = .510). Higher levels of T1 PUF were associated with higher levels of T2 DEB (*β* = .10, 95% CI = [< .001, .014],* p* < .001). Higher levels of T1 PUF were associated with higher levels of T2 body dissatisfaction (*β* = .08, 95% CI = [< .001, .06],* p* = .009). Higher levels of T1 ED were associated with higher levels of T2 body dissatisfaction (*β* = .18, 95% CI [.71, .81],* p* < .001). Higher levels of T1 body dissatisfaction were associated with higher levels of T2 body dissatisfaction (*β* = .08, 95% CI = [.01, .14],* p* = .013). All significant associations between DEB and PUF had weak effect sizes.

Among women, higher levels of T1 DEB were associated with higher levels of T1 PUF *(r* = .20, 95% CI [.14, .26],* p* < .001). Higher levels of T1 DEB were associated with higher levels of T1 body dissatisfaction *(r* = .55, 95% CI [.51, .58],* p* < .001). Higher levels of T1 PUF were associated with higher levels of T1 body dissatisfaction *(r* = .18, 95% CI [.13, .23],* p* < .001). Higher levels of T2 DEB were associated with higher levels of T2 PUF *(r* = − .06, 95% CI [-.11, .004],* p* = .029). Longitudinally, T1 PUF was not significantly associated with T2 PUF (*β* = − .03, 95% CI [− .07, .01],* p* = .133). T1 DEB was not significantly associated with T2 PUF (*β* = .06, 95% CI [− .02, .15],* p* = .184). Higher levels of T1 PUF were associated with higher levels of T2 DEB (*β* = .18, 95% CI = [.12, .24], *p* < .001). T1 DEB was not significantly associated with T2 DEB (*β* = .026, 95% CI [− .03, .09],* p* = .411). T1 body dissatisfaction was not significantly associated with T2 PUF (*β* = .01, 95% CI [− .05, .07],* p* = .714). T1 body dissatisfaction was not significantly associated with T2 DEB (*β* = − .03, 95% CI [− .09, .03],* p* = .270). Higher levels of T1 PUF were associated with higher levels of T2 body dissatisfaction (*β* = .06, 95% CI [< .001, .12],* p* = .047). Higher levels of T1 DEB were associated with higher levels of T2 body dissatisfaction (*β* = .15, 95%CI [.08, .22],* p* < .001). Higher levels of T1 body dissatisfaction were associated with higher levels of T2 body dissatisfaction (*β* = .17, 95% CI [.11, .23],* p* < .001). All significant associations between DEB and PUF had weak effect sizes. Although our longitudinal analysis provided evidence of directionality, it is important to emphasize that we cannot draw conclusions regarding causal relationships between the study variables.

**Fig. 1:**
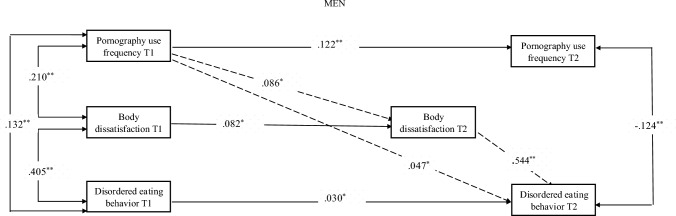
Mediation model regarding the mediating role of body dissatisfaction in the relationship between pornography use frequency and disordered eating behavior among men. *Note.* One-headed arrows represent standardized regression weights. Two-headed arrows represent standardized correlations. The dotted line presents the mediating role of body dissatisfaction. T1 represents the first data collection wave and T2 represents the second data collection wave, *p < 0.05; **p < 0.01

**Fig. 2:**
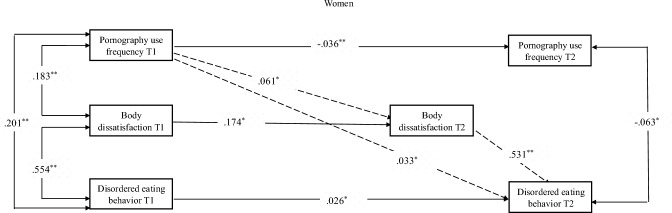
Mediation model regarding the mediating role of body dissatisfaction in the relationship between pornography use frequency and disordered eating behavior among women. *Note.* One-headed arrows represent standardized regression weights. Two-headed arrows represent standardized correlations. The dotted line presents the mediating role of body dissatisfaction. T1 represents the first data collection wave and T2 represents the second data collection wave, *p < 0.05; **p < 0.01

According to Model E’s findings, body dissatisfaction mediated the associations between T1 PUF and T2 BED among both men (*b*_indirect_ = .047, 95% CI [.012, .078], *p* = .011) and women (*b*_indirect_ = .033, 95% CI [.001, .050], *p* = .044). Thus, PUF predicted increases in body dissatisfaction which, in turn, predicted increases in DEB overtime among men and women as well. This indirect association was small and stronger indirect effects emerged via the autoregressive effects of PUF and DEB. Thus, body dissatisfaction partially mediated the relationship between T1 PUF and T2 DEB. The cross-lagged results, incorporating body dissatisfaction as a mediator, are presented in Figs. 1 and 2, and the indirect effects are presented in Table 5.

**Table 5:** Parameter estimates for cross-lagged panel mediation model of pornography use frequency, body dissatisfaction and disordered eating behavior

					*β*	b	*p* value
*Men*							
Direct effect							
Pornography use frequency T1	→	Disordered eating behavior T2			.101	.009	.000
Disordered eating behavior T1	→	Pornography use frequency T2			.023	.354	.510
Indirect effect							
Pornography use frequency T1	→	Body dissatisfaction T2	→	Disordered eating behavior T2	.047	.004	.011
*Women*							
Direct effect							
Pornography use frequency T1	→	Disordered eating behavior T2			.182	.019	.000
Disordered eating behavior T1	→	Pornography use frequency T2			.060	.369	.510
Indirect effect							
Pornography use frequency T1	→	Body dissatisfaction T2	→	Disordered eating behavior T2	.033	.003	.047

T1 represents the first data collection wave and T2 represents the second data collection wave

## Discussion

Previous studies examining the association between PUF and DEB include several limitations (e.g., use of cross-sectional study designs, focus on only men participants; Duggan & McCreary, 2013; Gewirtz-Meydan & Spivak-Lavi, 2023; Griffiths et al., 2018), leaving significant knowledge gaps in the literature. This study aimed to fill these existing gaps by investigating the cross-sectional and longitudinal associations between PUF and DEB among a young adult sample, considering potential gender-based differences. The present study also contributes to the literature by considering the body dissatisfaction as a mediating variable in the associations between PUF and DEB overtime.

The present findings suggest that men scored higher on PUF than did women. However, women scored higher on body dissatisfaction and DEB than men. The observed greater level in PUF could be attributed to men having higher solitary sexual drive (Dosch et al., 2016; Frankenbach et al., 2022; Oliver & Hyde, 1993) or men adopting more emotion-focused coping mechanism than women due to having difficulty in expressing their emotions (Lotfi-Hajilo et al., 2017; Tamres et al., 2002). The reason for the greater DEB and body dissatisfaction in women may drive from the tendency of women to attribute a greater significance to their physical appearance (Hudson et al., 2007; Öberg & Tornstam, 1999; Quittkat et al., 2019). These results are consistent with previous studies showing that men generally have greater levels of PUF (Dawson et al., 2020; Grubbs et al., 2022; Rissel et al., 2017), whereas women usually show greater levels of DEB and body dissatisfaction than men (Frederick & Essayli, 2016; Hudson et al., 2007; Quittkat et al., 2019).

Concerning the cross-sectional associations between the study variables, among men and women, T1 PUF and T1 DEB were positively associated with each other. These findings were consistent with previous studies reporting the positive association between PUF and DEB (Duggan & McCreary, 2013; Gewirtz-Meydan & Spivak-Lavi, 2023; Griffiths et al., 2018). However, T2 PUF and T2 DEB were negatively associated with each other. The negative correlation between PUF and DEB at T2 is noteworthy, particularly considering the positive significant association observed between T1 PUF and T2 DEB in the cross-lagged analysis. These differences may be attributed to the characteristics of cross-lagged models, which control for correlations within time-points and stability, across time. Therefore, if DEB remained stable, a simple correlation coefficient at T2 may not capture the true longitudinal association, as baseline levels (T1) would account for a significant portion of the variance later (T2) (Kearney, 2017).

Regarding longitudinal results, the present findings were mostly supportive of the first hypothesis as higher levels of baseline PUF were associated with higher DEB 1 year later with significant gender differences, but not the other way around, although these associations were weak. This finding suggests that PUF may be considered a risk factor for the development of DEB. This association was stronger for women than men. This finding is novel as previous studies did not include female samples. Yet, this finding is also consistent with The Self-Objectification Theory (Fredrickson & Roberts, 1997), which suggests that DEB may be more prevalent among women than men, as women’s bodies are more frequently objectified in society. Women may also be more sensitive to societal influences such as media and peer influences resulting in greater DEB symptoms (Thompson et al., 2004, Eisenberg et al., 2011). Moreover, significant positive association between PUF and DEB is in accordance with previous studies, which showed that greater engagement in pornography use may be linked to greater DEB (Duggan & McCreary, 2013; Gewirtz-Meydan & Spivak-Lavi, 2023; Griffiths et al., 2018). However, when comparing the present results to those of previous studies, it is crucial to consider their limitations, such as their sole focus on men. It is important to note that this is the first longitudinal study examining the associations between PUF and DEB, shedding light on the directionality of the associations between PUF and DEB among men and women as well.

PUF not only directly predicted DEB 1 year later, but also indirectly predicted it through body dissatisfaction, supporting the third hypothesis. The findings of the present study corroborate The Tripartite Influence Model (Thompson et al., 2004) and Social Comparison Theory (Festinger, 1954), which highlight that individuals might be influenced by sociocultural factors including peers, media, and family members, and as a result, individuals may tend to evaluate themselves against them when there is no objective reference for the evaluation. There are two main types of comparison: upward (comparing the self to better off) and downward (comparing the self to worse off). Given that pornography includes idealized bodies, it is more likely for the individual to engage in upward comparison and as a result one may develop body dissatisfaction, which, in turn, may result in eating problems (Gewirtz-Meydan & Spivak-Lavi, 2023). The gender differences in the associations between PUF and DEB may be attributed to various factors. For instance, women tend to be more sensitive to appearance ideals (Bornioli et al., 2021), and this sensitivity may be exacerbated by the prevalence of thin bodies in pornography. Such exposure can reinforce unrealistic body ideals, increase body dissatisfaction, and ultimately contribute to greater engagement in DEB among women.

Taken together, the present study has significant implications for both research and practical applications. In terms of research, it is necessary to conduct further studies focusing on the underlying mechanisms resulting in DEB, including diverse samples (e.g., not only men). Qualitative studies, such as the grid elaboration method (GEM), can be conducted to gain a deeper understanding of the association between PUF and DEB (Joffe & Elsey, 2014). With regard to practical applications, findings suggest that mental health professionals should consider incorporating strategies for addressing pornography use along with existing interventions for eating-related problems. Additionally, mental health professionals may develop treatment plans that include therapeutic techniques aimed at improving eating-related disorders, while also addressing pornography consumption and body dissatisfaction as they might be intertwined.

### Study Limitations and Future Directions

The present study has several strengths, one of which is the examination of the mediating effect of body dissatisfaction over time using large samples of both men and women. This addresses a gap in the literature, as previous studies have primarily been cross-sectional and have focused mainly on heterosexual and sexual minority men (Duggan & McCreary, 2013; Gewirtz-Meydan & Spivak-Lavi, 2023; Griffiths et al., 2018). However, this study is not without limitations. First, despite the longitudinal nature of the study, causal associations between the study variables cannot be established. Second, although previous studies have shown a link between PUF and DEB in sexual minority individuals, sexual orientation was not assessed in the present study (Duggan & McCreary, 2013; Gewirtz-Meydan & Spivak-Lavi, 2023; Griffiths et al., 2018). Third, the data collection for T2, but not T1, was conducted during the COVID-19 pandemic, and we observed a decline in PUF from T1 to T2. However, previous longitudinal studies have shown that changes in pornography use among adults (Grubbs et al., 2022) and adolescents (Bőthe et al., 2022) were only temporary, suggesting that these changes should not significantly impact our findings. Fourth, we did not provide a definition for pornography, potentially introducing some biases. Fifth, DEB scores were highly skewed, potentially due to the population’s characteristics (e.g., the sample was drawn from the general population, where eating disturbances have a relatively low prevalence rate) (Tury et al., 1994; Túry et al., 2021). Sixth, the percentage of explained variance was small varying between 3 to 7%. In this study, we used the SCOFF scale, although it demonstrates some degree of validity for screening eating disorders in the general population, its application as a diagnostic tool is uncertain. Nevertheless, it is recognized that SCOFF questionnaire captures eating disturbances rather than eating disorders. Seventh, our results were based on self-reported data, which may introduce some biases (i.e., recall bias or under- or over-reporting; Štulhofer et al., 2022). Finally, given that loneliness can influence both PUF and DEB (Cardoso et al., 2022), it would have been ideal to control for its potential effects through marital status. However, marital status varied between T1 and T2, and a substantial amount of missing data at T2 could not be adequately addressed using FIML. Therefore, we did not include marital status as a control variable. Future studies investigating the associations between pornography use and DEB should control for marital status, include sexual and gender minority individuals, and consider them in the statistical analyses. Additionally, future research may explore which specific types of DEBs (e.g., binge eating, anorexia, bulimia) are associated with PUF.

### Conclusion

Although previous studies have observed positive links between PUF and DEB, they had several limitations (e.g., lack of longitudinal findings, no data on women) that hindered the conclusions that could be drawn from their findings. To address these limitations, we investigated cross-sectional and longitudinal associations between PUF and DEB in a sample of adult men and women. The findings suggest that higher levels of PUF were associated with higher levels of DEB over time, with body dissatisfaction partially mediating this association among both men and women, although the observed effect sizes were weak. Based on these findings, clinicians treating clients with DEB may consider inquiring about their clients’ pornography use and body dissatisfaction as a potential factors contributing to the development and maintenance of DEB.

## Data Availability

The data analysis for the present work are available at the following OSF link (https://shorturl.at/NqcMc).
